# An ultrasound protocol for temporomandibular joint in juvenile idiopathic arthritis: a pilot study

**DOI:** 10.1259/dmfr.20200399

**Published:** 2021-07-08

**Authors:** Ingrid Tonni, Andrea Borghesi, Silvia Tonesi, Giulia Fossati, Francesca Ricci, Luca Visconti

**Affiliations:** 1 Orthodontics, Dental School, Department of Medical and Surgical Specialties, Radiological Sciences and Public Health, University of Brescia, Brescia, Italy; 2 Department of Radiology, Medical School, University of Brescia, Brescia, Italy; 3 Paediatric Immunology and Rheumatology Unit, University of Brescia, Brescia, Italy

**Keywords:** Juvenile Idiopathic Arthritis, Ultrasound, Temporomandibular joint, Children, Lateral peri-articular width

## Abstract

**Objectives:**

As it is well known, the diagnosis of temporomandibular joint (TMJ) involvement in patients affected by Juvenile Idiopathic Arthritis (JIA) is important to avoid the impairment of mandibular growth. In this context, Magnetic Resonance Imaging (MRI) is the gold-standard for detection of TMJ involvement, however it is expensive and requires patients’ collaboration. The aim of this study was to evaluate if ultrasound may be used as an alternative tool to investigate the acute signs of TMJ involvement in JIA patients.

**Methods:**

Lateral periarticular space (LPAS) and joint effusion were evaluated by ultrasound in a study Group A of 8 JIA children (11.6±3.5 years old) with 14 TMJs involved, as confirmed by MRI, and in a control Group B of 7 healthy children (9.3±1.2 years old) without temporomandibular disorders (TMD). The LPAS width values were compared between the two groups using the Mann–Whitney test. The ultrasound images of the JIA group were then matched with the corresponding MR images; the Spearman Rank Correlation test and the Bland–Altman test were used to evaluate the differences.

**Results:**

The LPAS values in Group A were statistically significantly higher than those in Group B (*p* < 0.001). There was no overlap of the LPAS values confidence intervals (CIs) between the two groups. No signs of joint effusion were identified in groups A and B. The Spearman test applied to the values of LPAS measured in ultrasound and the corresponding MR images showed a proportional positive correlation with a ρ of 0.623 and a *p* < 0.05.

**Conclusions:**

Ultrasound can detect differences in the TMJ features between JIA patients and healthy patients and it might be used as a follow-up tool in the assessment of TMJ involvement in subject affected by JIA.

## Introduction

Juvenile idiopathic arthritis (JIA) is the most common inflammatory rheumatic disease of childhood (1 in 1000 in the world). This inflammatory rheumatic disease is characterised by a chronic inflammation of one or more joints, with an onset before the age of 16 years and a minimum duration of 6 weeks.^1^ The involvement of the temporomandibular joint (TMJ) is quite frequent with a percentage from 17 to 87%.^2^

In short, an early feature of TMJ involvement is the synovitis, which is defined as a thickened synovia and is an active inflammation of the tissues. This inflammation can proliferate differently and with time leads to a chronic manifestation of JIA called pannus^3^, which is the quiescent form of the disease. Before this point is reached acute inflammation can cause severe sequelae in developing age individuals, such as destruction of the mandibular condyle and impairment of mandibular growth.^4,5^ Unfortunately, pain is a rare symptom of TMJ involvement and frequently the clinical assessment of this joint is not enough to formulate a diagnosis.^6^

In this context, JIA patients require an early diagnosis and frequent instrumental follow-up to evaluate the involvement and the progression of the pathology at the temporomandibular level. Currently, the gold-standard for detection of TMJ involvement in JIA is the Magnetic Resonance Imaging (MRI). This imaging technique is able to detect the signs of the acute phase of TMJs involvement such as the presence of synovitis, which is better demonstrated by contrast-enhanced (CE) MRI,^3,6^ joint effusion and bone marrow oedema. It also can detect the signs of the chronic phase of TMJs involvement such as condylar changes, bone erosion and abnormalities in form and position of the disc.^5^

However, MRI is an expensive examination and requires patients’ collaboration; it is lengthy with an imaging protocol time of approximately 45 min. It sometimes requires an intravenous administration of contrast agent and has some restrictions such as pacemaker or claustrophobia.^6–8^ As a result ultrasound could be used as an alternative in the assessmnet of TMJs involvement in pazients affected by JIA. This is because it is an easily accessible, low-cost, rapidly executing and non-invasive imaging technique with zero biological cost because of the absence of ionising radiation^5^.

For instance, the first article on ultrasound as a diagnostic tool to detect temporomandibular disorders (TMD) was published by Emshoff, Bertram, Rudisch and Gassner^9^, where they discussed the diagnostic value of ultrasound in determining the position of the articular disc in a population of adults with TMD. In the last 20 years US has been studied for the evaluation of increased thickness of the lateral peri-articular space,^2,4,7,10–14^ joint effusion,^2^ bone alteration such as bone erosion, condylar flattening and presence of osteophytes^2,4,7,11,12,15,16^ and disc dislocations.^7,11–13,15,17–20^

However, the review by Hechler, Phero, Van Mater and Matthews^5^ which investigated the performance of US compared to MRI in the detection of acute and chronic TMJ alterations in JIA patients, found a wide variation in the sensitivity ranging from 0–72% and specificity ranging from 70 to 83%. Further in one of the studies of the review by Hechler, Phero, Van Mater and Matthews^5^ the ultrasonography-assessed capsular width was compared with the MRI-assessed amount of synovitis,^10^ but the increase of the capsular width, which was observed with US, could indicate both the acute form (synovitis) and the chronic form (pannus) of TMJ involvement.

Additionally, the US studies with JIA children are limited in number and also heterogeneous for study design, parameters considered and the US protocol used. They do not have a clear distinction between the acute and chronic signs of TMJ involvement, such as to enable a univocal and therefore shared diagnosis of the involvement. They show differences in the frequency of the US probes and the US acquisition modes used. Further most of them do not carried out a direct comparison with MR images and do not have a control group.

Taking into consideration all the above cited aspects, a pilot study was considered needed: (1) to describe in detail the way US is used as a tool to investigate the TMJ in JIA patients, (2) to investigate if acute signs of TMJ involvement, such as increased peri-articular space and joint effusion, can be evaluated with US and (3) to overtake the difficulties of finding a control group with a negative diagnosis of TMD obtained by MR images.

The aim of this study, which will be followed by a longer one with a higher sample size, was to establish whether ultrasound could detect differences in TMJ features between JIA patients and a healthy group and it might be used as a tool to investigate the acute signs of TMJ involvement in JIA patients.

## Methods and materials

This study was conducted in accordance with the Declaration of Helsinki. It was approved by the Ethical Committee of Spedali Civili, Brescia (Italy). on the 9 October 2017, with a protocol study number 2831.

In this pilot, the ultrasound images of TMJs in JIA patients and in healthy patients were compared to identify differences in the width of the lateral periarticular space (LPAS) and in the presence of joint effusion. The features found in the ultrasound images of JIA patients were then compared with the findings in the corresponding MR images.

### Study group and controls

The study group A included eight patients diagnosed with JIA using the ILAR 2001 diagnostic criteria.^1^ The JIA children presented with 14 TMJs affected by the arthritic process as confirmed by enhancement presence in CE-MRI. The control group B included 14 TMJ without TMD in 7 healthy children.

The inclusion criteria of the Group A were: patients affected by JIA, with or without pharmacological treatment and absence of other systemic disease. The inclusion criteria of the Group B were: absence of TMD, no pharmacological treatment and absence of rheumatic and systemic diseases. The inclusion criteria common in both groups were: age less than 16 years and absence of previous or in progress orthodontic/gnathological treatments.

The patients in Group A were enrolled at the Dental Clinic of Spedali Civili, Brescia and they were referred to our centre by the Paediatric Rheumatology department of the same hospital. The patients in Group B were recruited from the Emergency Department of the Dental Clinic, Spedali Civili, Brescia. All parents and children were informed about the study procedures and a written consent was obtained.

### Procedures (Groups A and B)

Information about medical and dental history and past and present pharmacological treatments was collected. Patients underwent a clinical examination that consisted of an extraoral (aesthetic facial; TMJ and muscular functional analysis) and an intraoral (static and dynamic occlusion) investigation. Pre-existing instrumental records, such as orthopantomography, laterolateral teleradiography and posteroanterior teleradiography were also evaluated. Ultrasound images acquisition was performed in the subjects of Groups A and B maximum a month after the clinical examination. JIA patients undertook also an MRI for the diagnosis of TMJs involvement within one week before the ultrasound. MRI examination was not indicated for the healthy subjects. At the end a digital clinical chart was filled out with all the data collected.

### Ultrasonography

A recent review found that high-resolution ultrasound (HR-US) has more sensitivity and specificity in the detection of TMJ alterations compared to low-resolution ultrasound.^5^ The ultrasound investigation was performed in this pilot using an ultrasound scanner (“MyLab70XVG-6150”, ESAOTE SPA^®^, Genoa, Italy) with a 15 MHz high-frequency linear probe. All HR-ultrasound examinations were performed by the same observer, a radiologist with 15 years of experience in head and neck ultrasound imaging. The observer was blinded to subject’s group origin (patient *vs* control), clinical signs, symptoms and MRI findings.

During the ultrasound examination, the patient was in a supine position with the head turned to the right to examine left TMJ and to the left to examine the right TMJ according to Emshoff, Jank, Rudisch and Bodner.^18^ The transducer was positioned against the patient’s skin on the preauricular region of both TMJs. Two different probe positions were employed: transverse (axial direction) and longitudinal, which was parallel to the mandibular ramus (coronal direction).^17^ The transducer was tilted to be parallel to the mandibular ramus in order to obtain an optimal visualisation of the articular structures. The imaging protocol included axial and coronal scans at closed- and open-mouth.^17^ Once each image was displayed on the monitor it was “FREEZED” and interpreted. For each patient, the ultrasound analysis lasted about 10 to 15 min.

All images were evaluated with respect to the dimensional increase of the synovial joint space and the presence of joint effusion, which were usually considered as signs of acute inflammation.^2,4,10,13^

**Figure 1.**
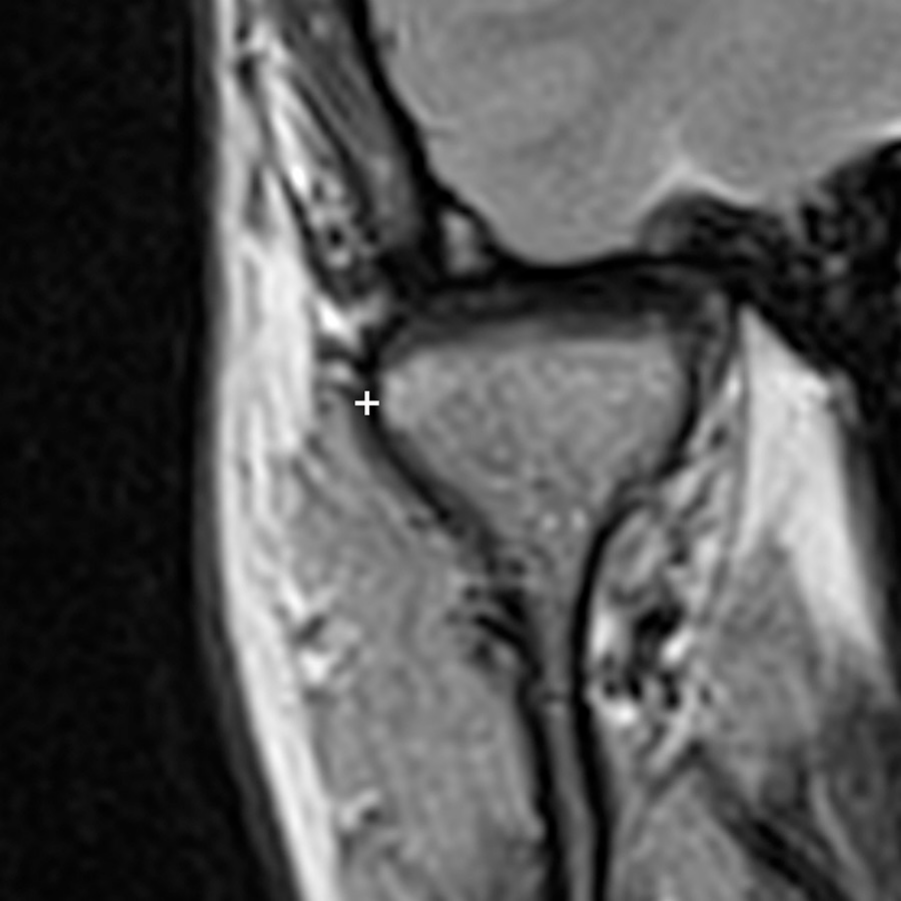
MRI of a right condyle in coronal view. The reference point on the condylar cortical (+) used to measure the LPAS is the most lateral cortical point at the condylar level. LPAS, lateral periarticular space.

**Figure 2.**
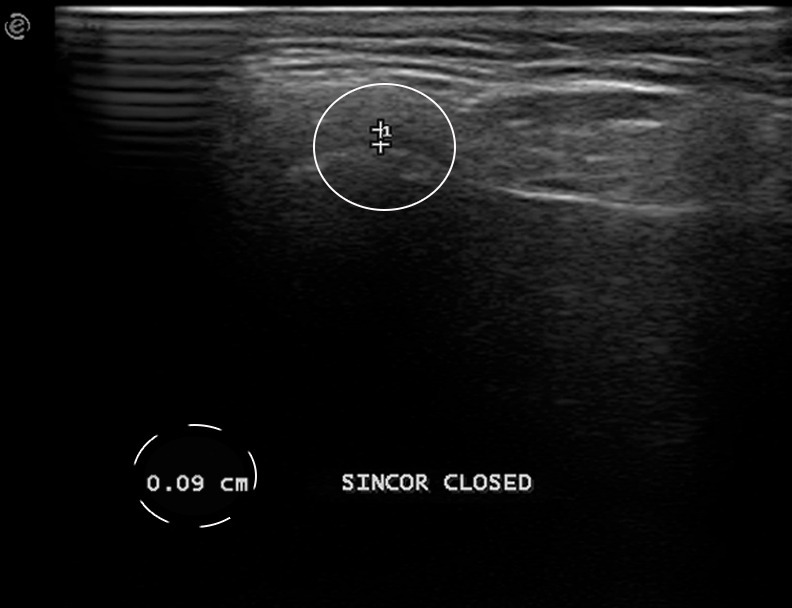
Ultrasound image of a left (SIN) temporo-mandibular joint. A linear probe in Coronal (COR) direction was used and the patient was in a closed-mouth position. The LPAS, which is the hypoechoic strip measured in orthogonal mode over the most lateral point on the condyle cortical, is identified between the two reference points (+). The width of the LPAS is 0,09 cm as showed in the dashed circle. LPAS, lateral periarticular space.

The width of the synovial joint space was measured from the cortical contour of the condyle to the contour of the capsule.^17^ The synovial joint space was defined as LPAS because the ultrasound examination allowed to inspect only the lateral area of the TMJ. The point of reference on the condyle to measure the LPAS was the most lateral one on the lateral cortical profile of the condyle head (Figure 1). The radiologist measured in ultrasound images the hypoechoic strip, which corresponds to LPAS, over the most lateral point on the condyle cortical in orthogonal mode both in the longitudinal and the transverse scans (Figure 2). The measurements were in centimetres.

The radiologist measured the LPAS on the ultrasound image in each acquisition mode providing four measurements of the right TMJ and four measurements of left TMJ. Two with the probe in Coronal and in Axial Closed-mouth position (CoC and AxC) and two with the probe in Coronal and in Axial Open-mouth position (CoO and AxO).

The presence of joint effusion was investigated in ultrasound images directly as a hypoechoic area within the articular space.^12^

### Contrast enhanced magnetic resonance imaging

**Figure 3.**
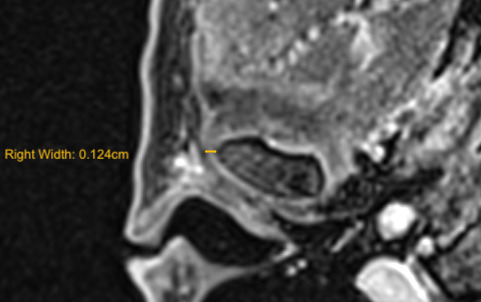
MRI of a right temporomandibular joint in the axial contrast enhanced Magnetic Resonance image (*T*_1_ weighted TSE).The LPAS width was measured from the reference point of the hypointense signal of the most lateral cortical of the condyle to the external limit of the periarticular tissue. LPAS, lateral periarticular space; TSE, turbo spin echo.

For this study, an MRI scanner with a 1.5T magnet (Magnetom Aera, Siemens, Erlangen, Germany) was used. The examination was performed before and after administration of a gadolinium-based contrast agent (Gadoteridol “Prohance” 0.2 ml/kgbw or Gadoteric Acid “Dotarem” 0.2 ml/kgbw). The post-contrast images were obtained in the axial plane using a fat sat *T*_1_ weighted turbo spin-echo (TSE) sequence (TR 553 ms; TE 12 ms; FOV 230; matrix 218 × 448; voxel size 0.5 × 0.5×3 mm; slice thickness 3 mm; scan time 2’28’’). The width of the synovial joint space was measured on the axial post-contrast images where the condyle has the largest cross-sectional area. The measurement of LPAS width was carried out using as reference point the hypointense signal of the most lateral cortical of the condyle until the external limit of the periarticular tissue (Figure 3).

### Statistical analysis

The TMJ was considered the statistical unit. The LPAS thickness in AxC, CoC, AxO, CoO and the average of the four measures, called AVG (AVERAGE), are continuous numerical variables. Joint effusion is a categorical variable. Descriptive statistics was applied for all TMJ measurements in both groups. Means, standard deviations (SDs), and confidence interval (CI) were calculated for continuous variables, and frequency (%) was used for the categorical variable.

To assess intraexaminer variability of the LPAS thickness and of joint effusion presence, the same observer repeated the ultrasound exam, measurements and observations twice in the same day. The interclass correlation coefficient (ICC) was applied for LPAS values and the Cohen’s κ for the presence of joint effusion. Dahlberg’s method was used to evaluate the measurement error.

The LPAS thickness in AxC, CoC, AxO, CoO and the average of these four measures AVG together with the CIs were compared between Group A and Group B with level of significance stated at α = 0.01 (x-2.58xSD/√n<=µ<=x+2.58xSD/√n). The Mann–Whitney test was used to assess the differences of the variables AxC, CoC, AxO, CoO and AVG between the two groups.

The Spearman Rank Correlation test and the Bland–Altman test were used to evaluate the difference between the values of LPAS in ultrasound and MRI. The significance level was set at α = 0.05. The analysis of data was performed using Jamovi (v. 1.6) and Microsoft Excel, v. 16.44.

## Results

### History and clinical examination

The Group A included 14 TMJs of 8 children (7 girls and 1 boy, mean age 11,6 ± 3,5 years, range 8,1–15,1 years). The Group B included 14 TMJs of 7 healthy children (4 girls and 3 boys, mean age 9,3 ± 1,2 years, age range 8,07–10,47 years). The mean age of all children at the time of examination was 10,5 ± 2,9 years.

The children included in Group A had the following JIA subtypes: oligoarthritic Antinuclear Antibodies+ (ANA+) in five children (62,5%), oligoarthritic ANA- in one child (12,5%), polyarthritis ANA- in one child (12,5%) and polyarthritis Rheumatoid Factor+ (RF+) in one child (12,5%). The pharmacological treatment was various: 25% of children were not in treatment, 25% were in treatment only with methotrexate, 37% with biologic drugs (such as Infliximab or Adalimumab) and 13% used only FANS (Naproxen). They presented one or more of the following signs and symptoms: altered opening/closing paths, reduced range of motion, painful joints or muscles, headache, eating problems. Two patients had only the right TMJ involved and six patients had both the TMJs involved, confirmed by Gd-MRI. The dental occlusion distribution was a Class II molar relationship in 75% of cases, a Class III molar relationship in 12,5% and a Class I relationship in 12,5%. The children included in Group B did not present any signs or symptoms of TMD or TMJ trauma at history and clinical examination. The dental occlusion distribution in Group B was a Class II molar relationship in 57% of cases, a Class III molar relationship in 14% and a Class I relationship in 29%.

### Ultrasound measurements of LPAS

The measurement error found with Dahlberg’s method was smaller than 0.02 cm. The intraexaminer reliability, which was evaluated with the ICC, resulted 0.861 for CoC, 0,538 for CoO, 0,641 for AxC and 0.509 for AxO showing a good reproducibility of the ultrasound findings. The LPAS thickness detected with the HR-ultrasound in the four acquisition modes (CoO, AxO, CoC and AxC) and the average of these four measures (AVG) together with the findings of the comparison between groups A and B are shown in Table 1. The LPAS values in Group A resulted increased in all the four different detections in comparison to those in Group B and the AVG value of LPAS in Group A was 0,086 cm compared with the AVG value in Group B that was 0,055 cm. The CIs (99%) of the LPAS values for the four different detections in Group A were not included in the corresponding CIs of Group B. The CI width of CoC values in Group A was the narrowest (0,022) of the four detections in the A group and this means that the CoC values were the most homogenous. The CIs width of the Group B was narrower than the CIs width in the Group A. Statistically significant differences (*p* < 0.001) were found with the Mann–Whitney test for the four variables CoO, AxO, CoC, AxC and the overage AVG.

**Table 1.** Temporomandibular joint LPAS thickness in groups A and B

	Group A (*n = 14*)				Group B (*n = 14*)					
	Mean	SD	CI (99%)	CI width	Mean	SD	CI (99%)	CI width	Mean diff.	*p* value
AxO	0,087	0,03	0,067; 0,108	0,041	0,054	0,005	0,050; 0,057	0,007	0,033	0.000^*a*^
AxC	0,087	0,018	0,075; 0,100	0,025	0,055	0,006	0,051; 0,058	0,007	0,032	0.000^*a*^
CoO	0,082	0,022	0,067; 0,097	0,03	0,055	0,006	0,050; 0,059	0,009	0,027	0.000^*a*^
CoC	0,089	0,015	0,078; 0,100	0,022	0,057	0,007	0,052; 0,062	0,01	0,032	0.000^*a*^
AVG	0,086	0,016	0,075; 0,098	0,023	0,055	0,004	0,052; 0,058	0,006	0,031	0.000^*a*^

AVG, Average; AxC, Axial closed; AxO, Axial open; CI, Confidence interval; CoC, Coronal closed; CoO, Coronal open; LPAS, lateral periarticular space; SD, Standard deviation.

Unit of measurement: cm.

a *p*<0.01.

**Figure 4.**
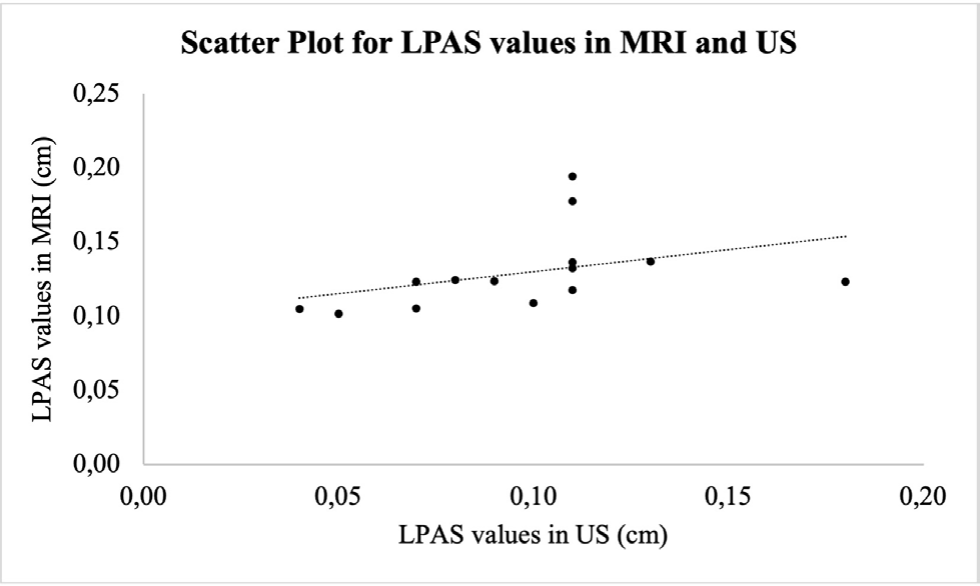
This scatterplot shows a moderate positive linear association between the LPAS widths in Ultrasound and the corresponding ones in MRI. LPAS, lateral periarticular space.

**Figure 5.**
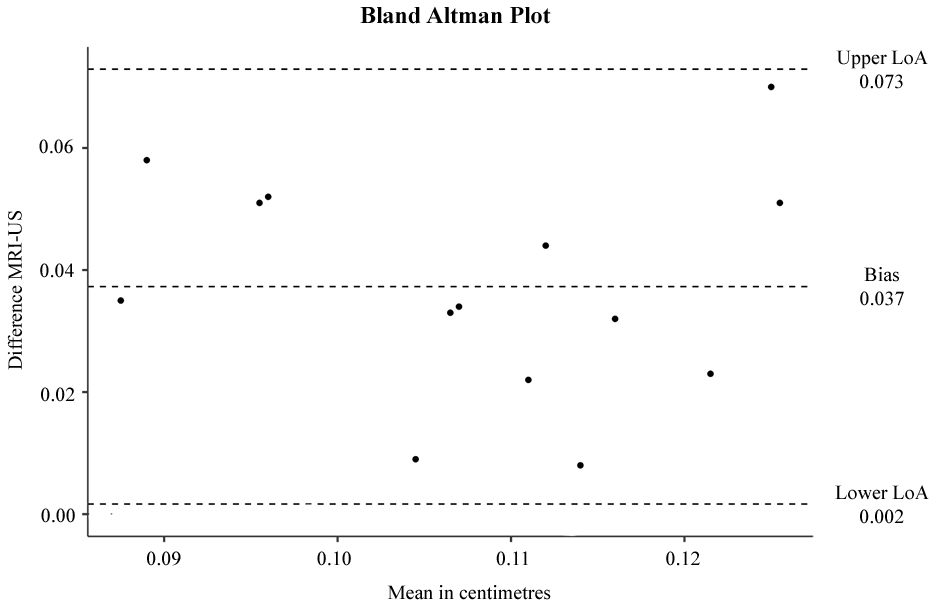
The graphical representation of the Bland–Altman Test results shows in abscissa the means in centimeters and in ordinate the difference of the measurements between MRI and ultrasound. The upper and lower LoA are 0.073 and 0.002 respectively. The mean of the differences is 0.037 cm but 95% of these differences are between the upper and lower limits. LoA, limit of agreement.

The Spearman test applied to the values of LPAS measured in ultrasound and the corresponding MR images showed a proportional positive correlation with a ρ of 0.623 and a *p* < 0.05 (Figure 4). The mean of the differences between the two types of measurements found with the Bland–Altman Test is 0.037 cm as shown in the graph (Figure 5). However, the distribution of the differences is normal, as indicated in Figure 5, where 95% of the differences are between the upper and lower limit.

**Figure 6.**
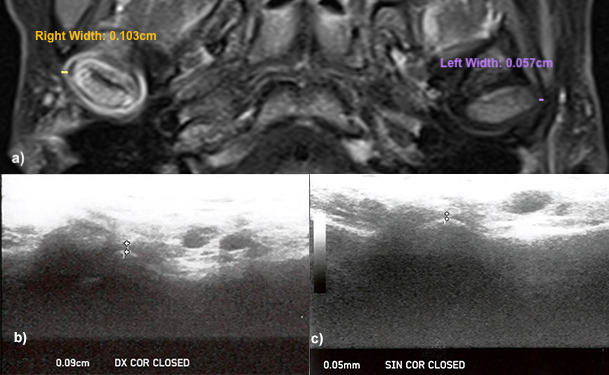
LPAS widths measured in MR and ultrasound images of a Juvenile Idiopathic Arthritis patient with acute involvement of the right temporomandibular joint. (**a**) Axial contrast enhanced MR image (*T*_1_ weighted TSE). The right temporomandibular joint presents a hyperintense signal (acute inflammation index), with increased LPAS width (0,103 cm). The left temporomandibular joint shows a lower LPAS width (0,057 cm), without acute inflammation. (**b, c**) Ultrasound images of the right (Dx) and left (SIN) temporomandibular joint. A linear probe in Coronal (COR) direction was used and the patient was in a closed-mouth position. Ultrasound images show a LPAS width of 0,09 cm in the right temporomandibular joint and a LPAS width of 0,05 cm in the left temporomandibular joint. LPAS, lateral periarticularspace; TSE, turbo spin echo.

**Figure 7.**
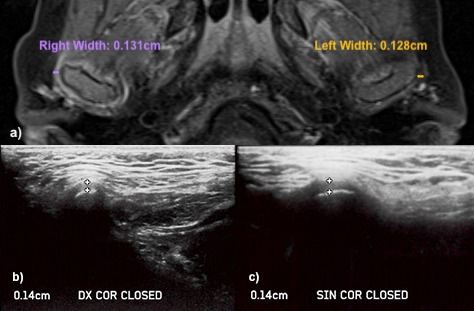
LPAS widths measured in MR and Ultrasound images of a Juvenile Idiopathic Arthritis patient with involvement of both temporomandibular joints. (**a**) Axial contrast enhanced MR image (*T*_1_ weighted TSE). The right temporomandibular joint presents a hyperintense signal (acute inflammation index), with increased LPAS (0,131 cm). The left temporomandibular joint shows an increased LPAS width (0,128 cm), without acute inflammation. (**b, c**) Ultrasound images of the right (Dx) and left (SIN) temporomandibular joint. A linear probe in Coronal (COR) direction was used and the patient was in a closed-mouth position. Ultrasound images show a LPAS width of 0,14 cm in the right temporomandibular joint and a LPAS width of 0,14 cm in the left TMJ. LPAS, lateral periarticularspace; TMJ, temporomandibular joint; TSE, turbo spin echo.

Some examples of the comparison between the LPAS values found in ultrasound and MR images are shown in Figures 6 and 7.

### Ultrasound qualitative report

Joint effusion was not detected with HR-ultrasound scans neither in subjects of Group A nor in subjects of Group B. The intraexaminer reliability evaluated with the Cohen’s κ (*k* = 1) showed a perfect agreement between the repeated ultrasound scans. Joint effusion was also no found in the MR images of the JIA patients.

## Discussion

The LPAS width and the presence of joint effusion were evaluated in JIA patients and in healthy patients in order to identify differences between the two groups. The ultrasound images of the JIA group were then compared with the corresponding MR images to investigate if ultrasound might be used as a tool to investigate the acute signs of TMJ involvement in JIA patients.

## Results

The difference between the AVG mean value of LPAS in groups A and B was 0,031 cm, and there was not overlap of the CIs for LPAS values in groups A and B (Table 1). The higher LPAS values found in US of JIA patients could indicate the increase width of the synovia, which is a characteristic sign of the arthritic TMJs in MRI and could be used as an ultrasound sign of TMJ arthritic involvement (Figures 6 and 7). However, the higher LPAS values do not give information on the acute or chronic nature of the TMJ involvement. The CI widths were lower in B, showing that the LPAS values of the healthy children were very similar. On the contrary they were larger in A and this could reflect the different TMJ involvement existing in the Group A, maybe related to the time of the JIA onset and to the different response that patients had to the therapy.

The LPAS mean values found in this pilot were lower compared to previous studies and this could depend on the condylar cortical level where the measurements were detected together with scan direction and patients’ age. In adult with normal TMJs, average values of 1.4 mm in longitudinal and 1.6 mm in transverse scans were reported.^17^ A capsular width cut-off value of 2 mm as an indirect marker of TMJ effusion in adults with rheumatic diseases or TMD was proposed by Manfredini, Tognini, Melchiorre, Bazzichi and Bosco^11^ and Manfredini, Tognini, Melchiorre, Zampa and Bosco.^21^ The results of studies in children showed that the cut-off level is lower than in adults. The TMJ capsular width in longitudinal scan of 68 children with JIA (mean age 11 years) was studied by Melchiorre, Falcini, Kaloudi, Bandinelli, Nacci and Matucci Cerinic.^13^ They showed that all 40 age- and sex-matched healthy controls without symptoms had an ultrasonography-assessed capsular width less of 1.4 mm and suggested a cut-off-level of 1.5 mm for JIA patients. However, they did not clarify the level of measurement on the condylar cortical. A cut-off-level of 1.2 mm ultrasonography-assessed capsular width measured in a longitudinal scan but at the subcondylar level in JIA children was proposed by Kirkhus, Gunderson, Smith, Flato, Hetlevik, Larheim.^10^

The result of the Spearman test (ρ: 0.623) indicates a moderate positive correlation between the values of LPAS measured in ultrasound and in MR images. This suggests that ultrasound might be used as a tool in the assessment of TMJ involvement in subject affected by JIA.

Some previous studies showed similar findings but there is not homogeneity regarding the signs detected in ultrasound images to make diagnosis of TMJ involvement in JIA patients.^5^ Different studies analysed different diagnostic parameters or combination of parameters of both acute (synovitis, joint effusion) and chronic inflammation (condylar erosion, condylar flattening, osteophytes and disc dislocations) in patients affected by JIA.^2,4,7,10,13,14^

The dimensional increase of the synovial joint space (identified also as “synovial width” “capsular width”, “thickening of the articular disc”, “peri-articular width”) is the parameter most analysed in the diagnosis of TMJ inflammation involvement.^2,4,7,10,13,14^ The area between the condyle and the capsule contains fluid, synovium and the disc, and the differentiation between those structures is difficult with ultrasound. An increase of the synovial width can indicate the presence of synovitis,^10^ the presence of joint effusion^2,4,13,14^ or the presence of disc displacement.^7^

In the present pilot study, the measurement of the synovial joint space width had the aim to identify synovitis or pannus considering that joint effusion and disc dislocations are less frequent and are always associated to synovitis, pannus or abnormal bone shape in JIA patients.^22^

The mean difference of 0.037 cm in the Bland–Altman plot can be explained by the fact that two different modalities (MRI and ultrasound) were used to measure the LPAS width, in addition the measurement plan was different in the two modalities (axial for MRI and coronal for ultrasound).

Joint effusion was never detected by ultrasound examination in both groups A and B. This is not in accordance with the previous literature where the presence of joint effusion was often detected in ultrasound images indirectly measuring the increase of the periarticular width.^2,4,13,14^ The low detection of joint effusion by ultrasound in this study could be related to the diagnostic criterium used to identify joint effusion, the observer evaluation and by the fact that the ultrasonographic image is limited to the lateral part of the joint. However, the MR examinations of all the JIA patients involved in this study did not show the presence of joint effusion, hinting that joint effusion as an MRI sign for the diagnosis of TMJ involvement by JIA should be questioned.

### Ultrasound protocol in patients with JIA

The time of images acquisition was 10–15 min, a tolerable time even for the youngest patients. Certainly, a much shorter time than that required for the MRI (35–45 min). Moreover, the ultrasound exam context was more comfortable for the patient compared with MRI for two other reasons. There was not need of the medium contrast injection and the young patients were more relaxed and more compliant as they had not remained closed in the hollow cylinder of the MRI for a long time.

In the present pilot, the linear probe was used on the TMJ region in two different positions (coronal and axial) and the TMJs of the patient were analysed with open and closed mouth in accordance with Elias, Birman, Matsuda, Oliveira and Jorge^17^ study. As a result from the pilot, the CoC position could be the only way to acquire ultrasound TMJ images in JIA and this was supported by the ICC for CoC (0.861) that shows a perfect agreement and by the narrowest CI width of CoC values in comparison with the other detection modes (CoO, AxO and AxC) in Group A (Table 1).

The coronal position resulted the most repeatable way to position the probe on the temporomandibular area during the pilot. Previously, Elias, Birman, Matsuda, Oliveira and Jorge^17^ proposed coronal scanning whenever the aim was measuring the lateral capsule–condyle distance. They observed that, during the production of the sonograms, variations in the measurements of the synovial width were more likely to occur with axial scanning, being much more dependent on the transducer tilting than the coronal scanning. The probe was only utilised in the coronal position to measure the periarticular space in JIA patients in the studies by Melchiorre, Falcini, Kaloudi, Bandinelli, Nacci and Matucci Cerinic^13^ and Kirkhus, Gunderson, Smith, Flato, Hetlevik, Larheim.^10^

In the present study, the radiologist preferred a closed-mouth than an open-mouth position because it was more stable and allowed to evaluate the joint structures for a longer time. Further, the closed mouth position, which was the only used by Kirkhus, Gunderson, Smith, Flato, Hetlevik, Larheim,^10^ allowed to be more precise in the measurement of the increased periarticular width.

The width of the synovial joint space is usually measured from the cortical contour of the condyle to the contour of the capsule. The cortical profile of the condyle is represented by a hyperechoic signal^13^ and the articular capsule presented as a hyperechoic line running parallel to the surface of the mandibular condyle.^11^ The synovial width can be measured at different levels on the condylar cortical: anterior and lateral level,^17^ subcondylar^10^ and condylar level.^2,4,10,11,13,14,17^

In this pilot study, the LPAS was identified as a hypoechoic strip over the most lateral cortical point of the condyle, at condylar level. This point is more easily identifiable and repeatable compared with the cortical points at the subcondylar or anterior level and this is very important for the frequent screenings and follow-ups of the TMJ involvement in JIA patients. On the contrary Kirkhus, Gunderson, Smith, Flato, Hetlevik, Larheim^10^ showed that ultrasonography evaluation of synovial thickening should be performed at the subcondylar level, because a laterally displaced disc may result in an altered capsular width at the condylar level. Further Elias, Birman, Matsuda, Oliveira and Jorge^17^ proposed to measure the articular space at the anterior level (distance between the most anterior point of the articular capsule and the most anterior point of the mandibular condyle) in an axial scan. However this measurement is particularly appropriate to judge the position of the disc and, as explained by Elias, Birman, Matsuda, Oliveira and Jorge,^17^ it should be enlarged in cases of anterior disc displacement.

### Criticism

The main limit of the pilot is that the exclusion of TMD in the Group B is based only on data collected during the clinical examination because an MRI would be not indicated in healthy patients.

Ultrasound examination is an operator-dependent procedure and another limitation of this pilot study is that the interexaminer variability of the LPAS thickness was not evaluated.

The Spearman test and the Bland–Altman test were applied to evaluate the values of LPAS measured in MRI and the corresponding ultrasound images, but the findings should be considered with caution because of the small sample size typical of a pilot study.

As already highlighted by Müller, Kellenberger, Cannizzaro, Ettlin, Schraner, Bolt^14^ contrast-enhanced MRI is the only procedure available to diagnose the JIA TMJ inflammation in its acute form. The periarticular width detected with the ultrasound could also be only expression of the pannus, which is considered a chronic alteration of the TMJ. It would be useful in future studies to find an indicator in ultrasound that matches only the acute inflammation in MRI.

## Conclusion

Ultrasound resulted a reliable tool to detect differences in LPAS widths between JIA patients and healthy patients. It might be used as a follow-up tool in the assessment of TMJ involvement in subject affected by JIA. The CoC could be the most appropriate acquisition mode for TMJ examination in JIA patients.
